# Three-Dimensional Printing of Bioinspired Hierarchical Structures for Enhanced Fog Collection Efficiency in 3D Space via Vat Photopolymerization

**DOI:** 10.3390/biomimetics9120734

**Published:** 2024-12-03

**Authors:** Daleanna Charoensook, Shah Md Ashiquzzaman Nipu, Ana Girish, Qingqing He, Shan Cheng, Kevin Chapman, Nathan Xie, Cindy Xiangjia Li, Yang Yang

**Affiliations:** 1 Department of Mechanical Engineering, San Diego State University, San Diego, CA 92182, USA; daleannacharoensook@gmail.com (D.C.); qhe7906@sdsu.edu (Q.H.); 2 School for Engineering of Matter, Transport and Energy, Arizona State University, Tempe, AZ 85287, USA; snipu@asu.edu (S.M.A.N.); agirish2@asu.edu (A.G.); 3 Canyon Crest Academy, San Diego, CA 92130, USA; shancheng017@gmail.com; 4 Westview High School, San Diego, CA 92130, USA; chapmank917@gmail.com (K.C.); nathanjingxie@gmail.com (N.X.)

**Keywords:** fog collection, biomimetics, hierarchical structures, 3D printing, vat photopolymerization

## Abstract

Collecting fog water is crucial for dry areas since natural moisture and fog are significant sources of freshwater. Sustainable and energy-efficient water collection systems can take a page out of the cactus’s playbook by mimicking its native fog gathering process. Inspired by the unique geometric structure of the cactus spine, we fabricated a bioinspired artificial fog collector consisting of cactus spines featuring barbs of different sizes and angles on the surfaces for water collection and a series of microcavities within microchannels inspired by Nepenthes Alata on the bottom to facilitate water flowing to the reservoir. However, replicating the actual shape of the cactus spine using conventional manufacturing techniques is challenging, and research in this area has faced a limitation in enhancing water-collecting efficiency. Here, we turned to 3D printing technology (vat photopolymerization) to create bio-mimetic fog collectors with a variety of geometric shapes that would allow for the most effective conveyance and gathering of water. Various barb sizes, angles between each barb in a single array, spine and barb arrangements, and quantity of barbs were tested experimentally and numeric analysis was carried out to measure the volume of water collected and optimize the mass rate. The result shows that optimal fog collection is with a mass flow rate of 0.7433 g/min, with *L_i_* = 900 μm, *θ* = 45°, *ϕ* = 90°, *N_b_* = 2, and *N_s_* = 5. This study presents a sustainable and ecologically sound method for efficiently collecting humid air, which is expected to be advantageous for the advancement of future-oriented fog-collection, water-transportation, and separation technologies.

## 1. Introduction

Water is an essential resource that plays a crucial role in human growth, as well as in promoting good health and overall well-being. However, currently, there is a prevalent scarcity of fresh water in arid and semi-arid regions. According to a 2017 assessment by the World Health Organization (WHO), at least two billion people lack access to safe drinking water and half of the world’s population may be residing in water-stressed regions by 2025 [1]. The water crisis results from global climate change, population increase, river siltation, larger water consumption, and the inadequacy of water collection and preservation in those regions [2,3,4]. Seawater desalination technology has become increasingly prevalent in the collection of freshwater in dry regions. Nevertheless, the exorbitant expenses and geographical constraints prevent its widespread promotion [5,6,7]. Fog is a form of atmospheric water vapor that constitutes approximately 10% of the collective capacity of all freshwater lakes on Earth, which has made it an attractive alternative to traditional water sources [8]. Moreover, a number of studies have demonstrated the critical importance of water fog collecting in semi-arid and dry environments, and it is theoretically feasible to collect substantial quantities of fog water with zero energy investment [9,10,11]. Over the course of billions of years, natural organisms have developed specialized surfaces that grant them unique capabilities, enabling them to thrive in challenging environments. Lotus flowers [12], water skippers [13], butterflies [14], desert cacti [15], spider silk [16], and desert beetles are just a few examples of the species whose surfaces exhibit wettability characteristics that aid in water collection. The Namib Desert beetle can effectively collect fog droplets from damp wind due to the presence of hydrophobic/hydrophilic patterned patches on its back [17]. Another example is Cotula fallax, a plant native to South Africa, which features tufts and leaves that are arranged in a hierarchical, three-dimensional pattern. Additionally, these leaves display hydrophobic properties that enable the plant to effectively gather water from fog towards its stem [18]. Also, several species of the Cactaceae family are capable of thriving in extremely dry desert environments by utilizing spines that gather fog from several directions [19,20,21]. Numerous conical spines with an apex angle of 2α cover the cactus’ surface. Also, conical barbs with an apex angle of 2β are located at the tip of each conical spine. These phenomena are primarily driven by the gradients of surface-free energy and Laplace pressure [22,23,24]. Insights from biological surface characteristics have sparked a new and exciting area of study: the creation of smart functional materials.

The interactions at the nano-scale, characterized by their structural simplicity and functional complexity, confer desirable and highly adjustable qualities to skills [25,26]. Several theoretical models concerning surface wettability have been formulated, such as Young’s contact equation [27], the Wenzel model [28], and the Cassie model [28]. The researchers elucidated the impact of surface roughness on the wetting behavior of water droplets on solid substrates. Certain fog collectors rely on gravitational or other external forces to convey or eliminate the accumulated water. However, the substantial dimensions necessary for water droplets to be propelled across a solid surface by the force of gravity obstruct the revival of the fog collection process [29]. Building upon the properties of spider silk and cacti, several researchers have employed gradient surfaces to tackle this issue [30,31,32]. For instance, to improve the effectiveness of water collection by condensation and fog harvesting, Gurera and Bhushan developed conical surfaces and triangular patterns to generate a pressure gradient and speed up the transit of water droplets. They went over how the surface area is the most important factor in how efficiently water is collected [33]. These surfaces enable efficient and uninterrupted fog collecting by utilizing the Laplace pressure gradient that is generated by the form and wettability gradient [34,35]. 

Various methods have been used to create microtips that resemble cactus spines, including chemical or electrochemical erosion of metal wire [36], deep X-ray etching [37], and replica molding [38,39]. However, the fabrication of complex 3D structures using traditional techniques remains difficult due to the limited ability of this approach to generate 2D surfaces with significant surface roughness and the larger production time. Three-dimensional printing is a highly effective method for creating customized parts with complex designs with a wide range of applications in various industries, academic settings, and everyday life [40]. It also opens up exciting possibilities for creating intricate structures inspired by nature [41,42,43]. Park et al. developed a textured hybrid surface by the application of a 3D copper oxide pattern over a flexible hydrophobic rough PDMS backdrop [44]. They found that the 3D bumpy hybrid surface exhibited a higher rate of water collection in comparison to the 2D flat hybrid surface. In addition, Koyun et al. employed a hybrid approach combining 3D printing and electrospraying to fabricate 3D surfaces with an exceptionally large surface area and a conical shape for collecting water from the atmosphere [45]. However, while successful in producing well-defined conical microtips and their arrays, there is still limited research on the directional transportation of collected water into reservoirs, which is crucial for practical applications. The bionic structure served as a significant source of inspiration within the laboratory. For example, Li et al. utilized submerged surface accumulation 3D printing to create the multibranched spines inspired by cacti [21]. Nevertheless, the printed spines exhibit noticeable stair steps because of the inherent characteristics of the layered additive manufacturing process. While the stair-stepping effect is advantageous for the condensation of fog, it would impede the downward dispersion of water droplets. To overcome this, Liu et al. employed expedited 3D printing to create hybrid structures for efficiently collecting fog water using a specialized printing technique. The results indicate that the printed spines with four longitudinal ridges exhibit the rate of fog collection [46]. However, the proportion rate of collected water is still a challenge. Furthermore, constructing multi-barbs arrays with a controlled configuration, like those seen in nature, is a difficult task. Achieving adjustable 3D geometric shapes in branched spine arrays, which can significantly enhance fog collecting efficiency, is challenging using these techniques [47]. 

To address the constraints of prior research, we introduced an artificial fog collector structure that imitates the structural characteristics of cactus spines by utilizing 3D printing technology to ensure a surface that effectively condense fog and collect water. To transport the collected water over the vertebral surface, a network of microchannels including microcavities, inspired by the pitcher plant Nepenthes Alata, was also developed. Moreover, the volume of water collected, and the mass flow rate were measured and optimized by testing different barb sizes, angles between each barb in a single array, spine and barb configurations, and quantity of barbs. Fog harvesting in areas with water scarcity could be achieved by the design and fabrication of a large-scale artificial continuous fog collector using this optimum technique.

## 2. Materials and Methods

### 2.1. Materials and Fabrication

The biomimetic micro-trichomes mimicking those found in cactus spines were fabricated using Digital Light Projection (DLP) 3D printing technology. Compared to other fabrication methods, 3D printing offers the structural versatility necessary for replicating the complex geometry of trichomes. Given that cactus spines consist of multiple barbs with varying sizes and angles, an additive manufacturing approach is ideal over conventional techniques, which may lack the precision and adaptability required for such intricate structures.

The Standard Triangle Language (STL) models of the cactus spines were uploaded into KUDO3D (v3.1), a slicing software, where they were processed into 2D pattern layers for the DLP printing process. The models were printed using a Kudo3D Micro printer with UHR resin from Kudo3D Inc. (Dublin, CA, USA). The DLP process cured the resin with a high resolution of 15 μm in the x–y plane and 10 μm in the z direction, ensuring fine detail and accuracy in the final printed structures.

### 2.2. Modeling of Cactus Spine and Microchannel

**Figure 1:**
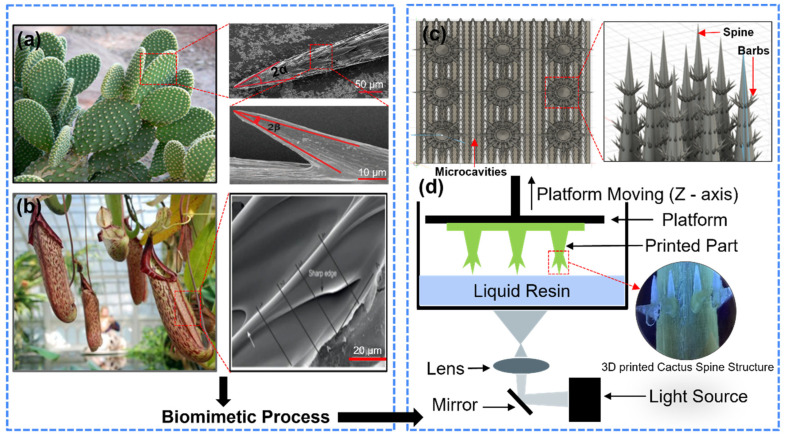
Biomimetic fog water collection system: (**a**) natural cactus structure with SEM image of conical spine and conical barb, (**b**) pitcher plants (Nepenthes Alata) with SEM image of microcavity within microchannel, (**c**) CAD model of bio-inspired water collection system with spine, barb, and microchannel structure; (**d**) photopolymerization 3D printing process with SEM image of 3D-printed object.

The design of fog collector inspired by the cactus Opuntia microdasys (Figure 1a,b) was developed by considering five primary factors: size (*L_i_*), the angle (*θ*) formed between adjacent barbs, hierarchical arrangement (uniform vs. alternating), number of barbs (*N_b_*), and spinal arrangement (*N_s_*). The sizes of the spines were varied, with seven distinct barb sizes ranging from 650 to 1150 μm (Supporting Information, Table S1); however, for laboratory testing, three specific sizes (900, 1000, and 1100 μm) were selected due to their relevance to the desired functional outcomes. Each barb was designed with varying angles and hierarchical configurations, including both uniform and alternating patterns. Additionally, the barb was created to include either one or two sub-barbs to explore the impact of these features on fluid collection performance. The spine surfaces were oriented perpendicular to the fluid flow and aligned in the +Z direction to maximize the gravitational force acting on the collected water. Microchannels and microcavities were designed to enhance fluid flow and minimize blockages with a pinning angle of approximately 15° and a spacing of 0.3 mm between each cavity to facilitate water transport and retention. The microchannels featured edges angled at 45° to the platform surface, which helped prevent water from accumulating and obstructing flow, while an array of micro-beams at the end of each microchannel further optimized fluid dynamics. The overall structural arrangement aimed to maximize water capture and retention in environments simulating natural fog conditions.

The design and modeling process utilized Autodesk Fusion 360, where each variation was carefully predesigned and optimized based on theoretical performance metrics (Figure 1c). The models were then exported as STL files and fabricated using the Kudo3D Micro printer, selected for its high precision (15 μm resolution and a *Z*-axis stepping size of 10 μm). The printing process involved slicing each model into 10 μm layers and printing in the +Z direction to ensure the highest degree of structural fidelity (Figure 1d). The material used for fabrication was Kudo3D 3DSR UHR resin, known for its mechanical properties, including a tensile strength of 29.2 MPa, Young’s modulus of 1284 MPa, a heat deflection temperature (HDT) of 52.7 °C, and a low shrinkage rate of 1.7%, which is crucial for maintaining the integrity of the fine structural details. Post-processing involved cleaning the printed samples in isopropyl alcohol with agitation to remove any uncured resin, followed by UV post-curing for 10 min using an Anycubic Wash & Cure station.

### 2.3. Testing Environment 

A controlled testing environment was created by fabricating an acrylic enclosure (Supporting Information, Figure S1). A digital hygrometer was mounted on the rear of the container and fluid flow intake was made possible by fabricating a circular extrusion on the side of the enclosure. At room temperature, a saturated water flow with a relative humidity of 95% and a maximum flow rate of about 4 mL/min was used to investigate the water collection capabilities. The weight of the collection cup was measured and recorded incrementally on the Ohaus scale over a total collection time of 60 min. At *t* = 60 min, the sample and the collection cup were weighed again on the same scale. A platform was also made to support the sample and allow for precise control over its orientation and placement in relation to the flow. 

### 2.4. Finite Element Analysis (FEA) of Cactus Structure

The fluid flow and condensation rate of cactus structure were analyzed using the COMSOL Multiphysics (6.1) simulation software. The model included velocity, pressure, and temperature profiles at *t* = 0, 15, 30, and 60 s. To begin, COMSOL Multiphysics was fed the two-dimensional (2D) cross-sectional geometry of the building modelled in SolidWorks. The calculation was made simpler by using a single cactus structure with a specific barb length (*L_i_* = 900 µm) and angle between adjacent barbs (*θ* = 45°), which was simulated with three distinct sub-barb angles *ϕ* = 45°, 75°, and 90° (on the XZ plane). With the use of a 2D transient computation model for fluid flow and the air moisture transport module, we mimicked the real-world conditions by simulating humid air using water vapor. The external environment’s fluid flow was configured as laminar flow, and the humid air and environment had a diffusion coefficient of 2.344 × 10^−5^ m^2^/s. 

## 3. Results and Discussion

### 3.1. Experimental Analysis

The fog water collection performance of the artificial cactus spine depends on its shape, wettability, and spine arrangement. Moreover, the efficiency of cactus-mimetic spines in collecting water is influenced by the rate of water condensation and the speed of water transportation [48]. The condensation of water is determined by the vapor pressure. Fast growth of the water droplet occurs when the origin (the initial stage of water droplet formation on the surface of the barbs during the condensation process) surpasses the critical size *r_c_*, and the vapor pressure is lower than the real pressure. Utilizing the Kelvin equation (Equation (1)) [49], we propose that including barbs of varying length and angle on the surface of the spines can offer more tips to capture fog and produce water droplets.

(1) $$P=e^{\frac{2\sigma_{w}V_{ml}}{RT}k}P_{s}$$

This equation represents the molar volume of water (*V_ml_*), the surface tension of water (*σ_w_*), the gas constant (*R*), the temperature (*T*), the saturated vapor pressure of water on a flat surface (*P_s_*), and the vapor pressure of water on a surface with curvature (*k*). 

The critical coagulate radius, *r_c_*, of water can be determined as follows.

(2) $$r_{c}=\frac{2\sigma_{w}V_{ml}}{RTln\left(\frac{P}{P_{c}}\right)}$$

In the preliminary phase of water condensation, the surface curvature (*K*) of the condensed water droplet can be approximated as the curvature of the solid−liquid interface. Consequently, augmenting the curvature of the solid−liquid interface would elevate the vapor pressure (*P*) and diminish the crucial condensation radius (*r_c_*). Both theories suggest that fog capture is more efficient near the points of the spine; hence, the formation of structures with more curvature would enhance fog collecting.

**Figure 2:**
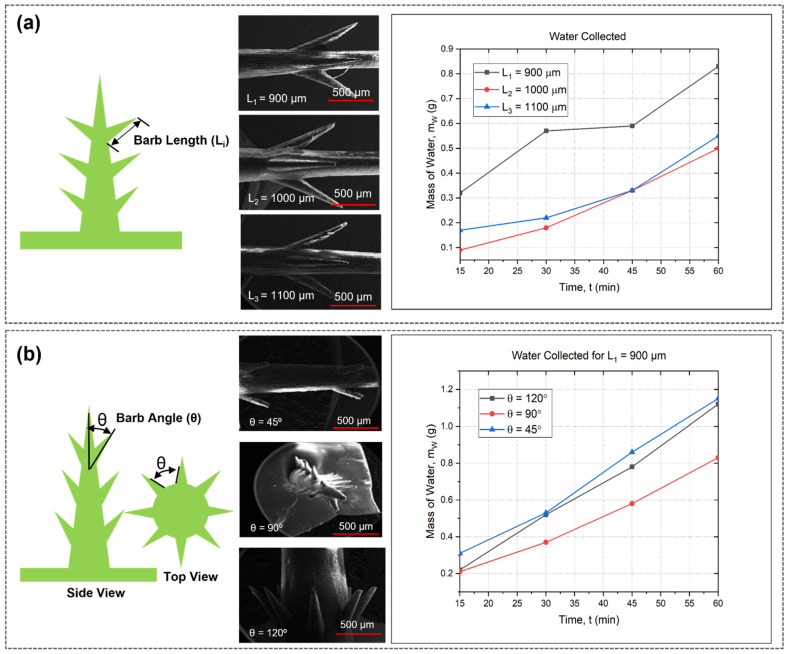
Water collection and transportation of 3D-printed cactus structure: (**a**) microscopic image of different barb lengths 900 µm, 1000 µm, and 1100 µm with water collection amount over time; (**b**) microscopic image of different barb angle 45°, 90°, and 120° with amount of collected water for barb length 900 µm over 60 min period.

The vapor pressure exhibited a positive correlation with the curvature k of the cactus spine tip. The water collection mechanisms of the 3D-printed spines were investigated by changing the angle and length of the barbs on the cactus spine. Figure 2 shows the surfaces’ water collection rates. It was found that the barb with a smaller length (900 µm) condensed more water droplets (0.83 g) after the period of 60 s (Figure 2a). At each instance, the water droplet condensing speed of the 3D-printed spines with a length of 900 µm was significantly higher at every interval than that of the spines constructed with a longer barb length. The observed pattern suggests that a smaller barb length improves the effectiveness of water collection because of a more advantageous surface-to-volume ratio and the capacity to efficiently capture and direct condensed water droplets [50]. A more prominent slope for *L*_1_ indicates a greater water capture rate, indicating that the 900 μm spines are more well-suited for the micro-scale interactions between fog droplets and the spine surface. In contrast, the larger barb sizes (*L*_2_ and *L*_3_) showed lower rates of water collection. This can be ascribed to a decrease in surface roughness and worse aerodynamic efficiency, which may result in less efficient capture or retention of droplets. Length has a major impact on the aerodynamic characteristics of the barb structures. To collect water droplets in the air and guide them to the surface, shorter barbs are superior at producing localized turbulence and blocking airflow. Conversely, longer barbs enhance drag and flatten the airflow surrounding the structure, decreasing the possibility of droplet capture. Longer barbs may also cause water droplets to be more deflected, which lowers the collecting rate.

Figure 2 also demonstrates the effect of variation in barb angle on the water collection rate. To measure the efficiency of each angle on barb sizes, we used a barb length of 900 µm (Figure 2b). The angle of adjacent barbs of the natural cactus shown in Figure 1a is approximately 40–45 degrees. This angle plays a crucial role in the cactus’s ability to effectively interact with its environment, and it served as inspiration for the design of our bioinspired structures. The result indicates that the cactus spine structure, characterized by a barb angle of 45°, has a higher water collection rate over time in comparison to structures with angles of 90° and 120°. The reduced barb angle of 45° facilitates effective adherence of water droplets to a greater surface area, hence promoting accelerated coalescence of smaller droplets into bigger ones [51]. The acute angle facilitates the formation of a more pronounced gradient, therefore enhancing the orientation of droplets towards the lower part of the structure, where they can aggregate more effectively. Conversely, broader angles (90° and 120°) decrease this gradient, therefore decelerating the transit of water droplets and resulting in less effective collection. Furthermore, a reduced barb angle amplifies capillary action, which refers to the capacity of a liquid to move across confined areas without recourse to external pressures. The reduced angle results in a more constrained capillary channel on the surface, therefore enhancing the downward displacement of water droplets by capillary forces [52]. Greater angles exhibit reduced capillary force because of a wider and more level surface, therefore impeding the efficient movement and accumulation of water. Furthermore, when the angle is set at 45°, gravity can even more efficiently facilitate the downward movement of water once droplets have attached to the surface. The more acute angle creates a straight trajectory for the water to flow due to gravitational attraction. At larger angles (90° and 120°), the plane of flow becomes more horizontal, leading to the possibility of droplets being trapped or undergoing re-evaporation instead of flowing downwards for collection. To consistently gather water on the apex of the 3D-printed spines, the compressed water droplet must advance down the spines that mimic cacti towards the base. The apparent direction of growth of the spines from the cactus stem did not seem to be a significant determinant in the directional migration of the water droplets. The water transport of spines resembling cacti is ascribed to the difference in Laplace pressure on two sides. The formula for representing the pressure differential is as follows (Equation (3)) [36].

(3) $$\Delta P_{curvature}=-\int_{0}^{L*tan\frac{\alpha }{2}}\frac{2\sigma_{w}}{{\left(R+R_{0}\right)}^{2}}sin\frac{\alpha }{2}dz$$

where ∆*P_curvature_* is the gradient of Laplace pressure, *L* is the length of the 3D-printed cactus-mimetic spine barb, *R* is the radius of curvature of the surface or the spine where water droplets form and *R*_0_ is the radii of the 3D-printed cactus-mimetic spine barb and the collected water droplet, α is the tip angle, and *dz* is the integral variable of the 3D-printed cactus-mimetic spine barb.

**Figure 3:**
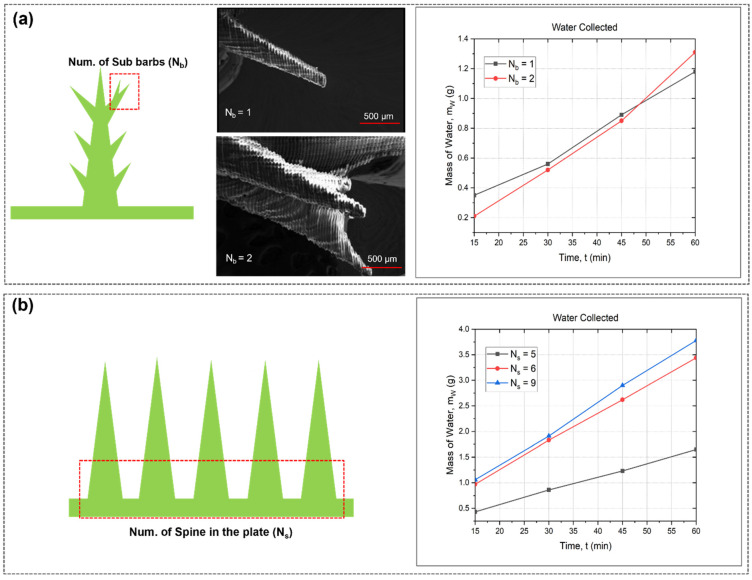
Water condensation system of 3D-printed bio-mimetic cactus structure: (**a**) microscopic image of barbs with water collection in masses (g) over time; (**b**) microscopic image of artificial cactus and comparison of spine number with water collection over time of 60 min period.

We also look at how different sub-barbs and spine counts affect the water condensation of 3D-printed artificial cactus structures. Prior research on water-collecting substrates and devices mostly concentrated on the single barb artificial spine array because of the constraints inherent in its production capacity. Cactus-mimetic multi-barb spines have the potential to greatly enhance water-collecting efficiency. Figure 3a illustrates the temporal accumulation of water mass by the 3D-printed cactus-mimetic spine structure with varying barb counts: *N_b_* = 1 and *N_b_* = 2. At the outset, the construction with a solitary barb (*N_b_* = 1) accumulates a little higher amount of water, reaching roughly 0.6 g after 20 min, in contrast to the double barb (*N_b_* = 2), which collects around 0.5 g. Over time, the performance of both structures becomes comparable as the 45 min mark approaches. However, by 60 min, the structure with two barbs outperforms the single barb by collecting the largest amount of water, approximately 1.3 g, while *N_b_* = 1 collects around 1.2 g, indicating that although a single barb may offer faster initial water collection, the presence of two barbs enhances the total capacity and efficiency in the long run. This is because the larger surface area for droplet adherence and improved coalescence among the barbs result in more effective water capture over extended periods of time. Moreover, the spines are anatomical structures derived from natural species, purposefully engineered to optimize the absorption of water from fog by augmenting the surface area and facilitating the generation and diffusion of droplets. The efficiency of the system in collecting water demonstrates a substantial improvement as the number of spines rises from 5 to 9, as evidenced by the progressive increase in the mass of water collected over time. The building with a spine count of nine constantly accumulates the most amount of water, reaching around 3.5 g after 60 min Figure 3b. This suggests that the architectural design or surface structure of the structure improves the efficiency of capturing fog. These findings indicate that an increased quantity of spines leads to a greater number of chances for water droplets to condense and merge, thereby improving the overall efficiency of water collection [53].

### 3.2. Simulation Analysis

**Figure 4:**
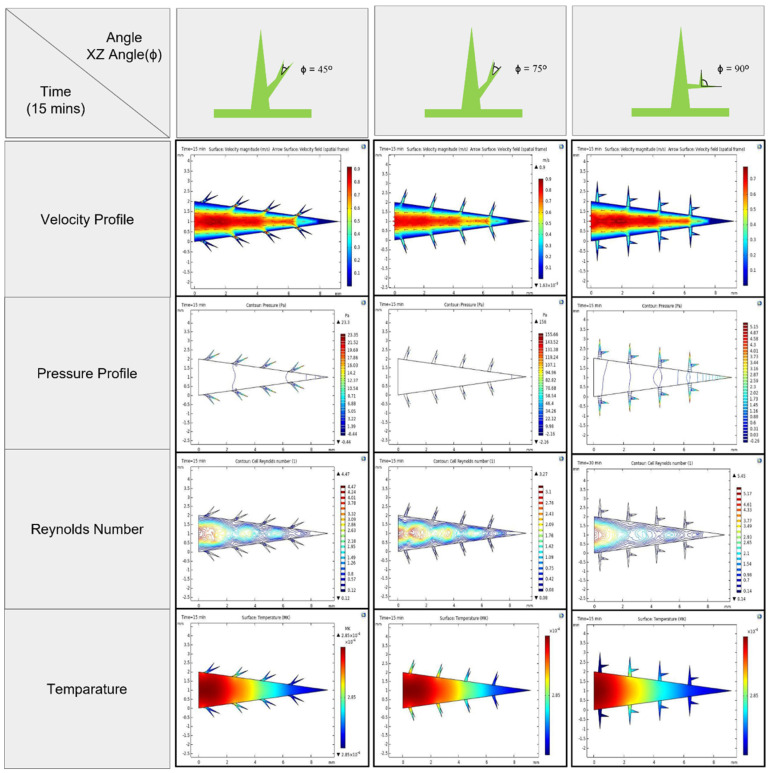
Bionic simulation of 3D−printed spine structures with the effect of four essential parameters (velocity, pressure, Reynolds number and temperature) at different barb angles (XZ plane) with fifteen−minute time interval.

Distinct configurations of our 3D-printed artificial cactus structure will provide turbulence in the flow stream, which will facilitate water collection. In this study, we simulated the impact of arranging multi-barb spine arrays by tilting and movement on the flow stream. The resulting flow stream around each branching spine is depicted in Figure 4. The included simulation graph evaluates the influence of several barb angle angles on the XZ plane (45°, 75°, and 90°) on the efficiency of a bioinspired fog collecting device. It especially investigates the effects of these angles on several parameters throughout a 60 min timeframe (Supporting Information, Figures S2–S5).

The given data encompasses four essential parameters: velocity profile, pressure profile, Reynolds number, and temperature distribution at 15 min time interval (Figure 4). These metrics enable a comprehensive understanding of the dynamics of fog accumulation at various barb angles. A velocity profile at an angle of 45° exhibits a streamlined airflow over the period of 60 min, characterized by a uniform distribution and less disturbances around the spine structure. The efficient transfer of fog droplets towards the tips, where they can be caught, is crucially facilitated by this streamlined flow. An analysis of the pressure profile at this angle reveals no pressure decreases, suggesting a consistent flow that facilitates uninterrupted fog movement across the collector’s surface. The residual Reynolds number stays rather low, indicating a regime of laminar flow. This flow pattern is typically beneficial for the purpose of collecting fog, since it reduces the separation of water droplets from the surface and enhances their attachment to the core structures. Moreover, the temperature distribution is extremely consistent over the surface, indicating that temperature gradients have minimal impact on the water-collecting operation. Therefore, it can be shown that a 45° angle has the potential to establish an optimal equilibrium between flow dynamics and surface interaction, improving the total efficiency of water collecting. By comparison, when the angle is set at 75°, the velocity profile shows very modest amounts of turbulence, as indicated by the presence of uneven airflows around the spines. This turbulence has the potential to augment the interaction between fog droplets and spines, hence potentially improving the rates at which droplets are captured. Nevertheless, the simultaneous pressure profile suggests heightened pressure fluctuations, which can result in elevated flow resistance and diminished collection efficiency because of droplet detachment. The greater Reynolds number at this angle indicates a shift towards a flow regime characterized by increased turbulence. Although a certain level of turbulence might enhance droplet capture by promoting contact with the surface, too much turbulence can disturb the flow and thereby decrease the overall efficiency. When the angle is 90°, the velocity profile exhibits substantial turbulence characterized by prominent eddies and chaotic flow patterns. This turbulence, characterized by a high Reynolds number, disturbs the flow of fog and raises the probability of droplet separation, therefore decreasing the effectiveness of cloud collecting. The pressure profile at this angle exhibits a significant amplification in pressure variations, hence exacerbating flow instability. The temperature distribution exhibits heterogeneity, which may impact the condensation and coalescence processes of water droplets. In summary, the findings indicate that a barb angle of 45° provides optimal conditions for fog collection by promoting streamlined flow, maintaining pressure stability, and supporting laminar flow dynamics. This configuration minimizes droplet detachment while enhancing droplet transport and adhesion. The micro-scale turbulence near the spines at 45° complements the uniform macroscopic flow, enabling efficient droplet interaction and maximizing water collection efficiency. Conversely, larger angles (75° and 90°) introduce excessive turbulence and flow instability, reducing the effectiveness of the fog collection system.

### 3.3. Analysis of Barb Arrangement 

**Figure 5:**
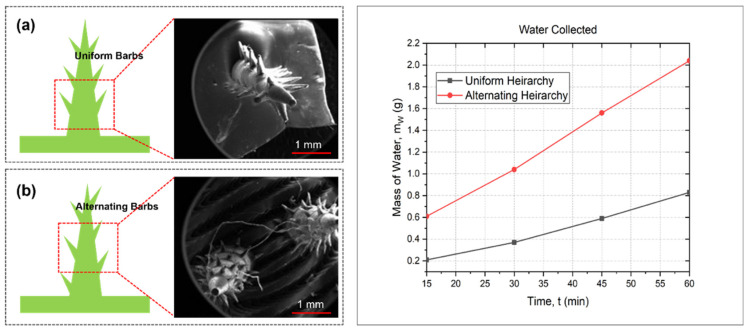
Comparison of barb arrangement for water condensation of 3D-printed artificial cactus spine structure: (**a**) microscopic view of uniform barbs; (**b**) microscopic view of alternating barbs.

The configuration of barbs also has a substantial impact on the process of fog gathering. All of our experiments and numerical analysis were conducted using a consistent barb layout. Furthermore, we conducted a comparison analysis between the uniform barb arrangement and the alternating barb arrangement to investigate the structure of the barb arrangement on water collection. Figure 5 illustrates the comparative examination of two hierarchical structures, “Alternating Hierarchy (AH)” Figure 5a and “Uniform Hierarchy (UH)”; Figure 5b shows a fog collecting system. The evaluation was conducted for a duration of 60 min. The results revealed that the “AH” has a greater ability to collect water, as shown by a higher rate of accumulation over the whole experiment period. During the first 15 min time interval, the “AH” attains a water mass of around 0.6 g, which is significantly higher than the 0.2 g collected by the “UH.” This pattern persists as the “AH” accumulates around 2.0 g of water after 60 min, whereas the “UH” reaches a plateau at around 0.8 g. The improved performance of the “AH” can be ascribed to its variability in surface properties, which can either augment the surface area available for condensation or raise the efficiency of water drainage. Conversely, the “UH” consistently maintains a linear but restricted rate of water collection, indicating that a surface with more uniformity does not enhance the physical processes necessary for effective fog harvesting. These results emphasize the possible advantages of incorporating alternating hierarchical structures in fog collecting system design to optimize water production.

## 4. Conclusions

In conclusion, we have successfully demonstrated an artificial fog collector inspired by cacti with various geometric structures and a system of microchannel cavities at the bottom to facilitate water flow to the reservoir. The three-dimensional printing technique made it possible, which clearly offers distinct advantages over other structured surfaces, to optimize fog collection by including both fog-capturing and water-transportation processes. The fundamental mechanism consists of the following components: firstly, the presence of a turbulent and complex flow field around the optimized spines enhances the effective deposition area, so enabling the deposition of smaller water droplets and finally facilitating water collection; secondly, the directional movement of water droplets on these surfaces significantly impacts their efficiency in collecting water. More precisely, water droplets on spines and barbs experience varying Laplace pressure on the two opposing sides. The tip of the cactus-inspired spine barb was adjusted according to its length and angle to facilitate rapid water movement along the spine. Current fog-harvesting methods have yielded around 200 L of water daily over the past year from a single 40 cm^2^ fog collector, resulting in a mass flow rate of 0.03472 g/min per mm^2^. The ultimate integrated fog collecting device of this study attains a mass flow rate of 0.07433 g/min per mm^2^, representing an 115% enhancement compared to the initial mass flow rate difference of 0.03961 g/min per mm^2^. Microchannels with microcavities are able to transfer more water to the bottom reservoir in the same length of time as other designs due to their directional water spreading capabilities, which enhance efficiency. Moreover, the efficacy of fog collecting after 3D printing can be enhanced experimentally by applying surface chemical treatments such as sputtering hydrophobic coating with a self-assembled monolayer [21] or dip-coating fluorodecyl polyhedral oligomeric silsesquioxane and poly (ethyl methacrylate) [54]. Unequivocally, the amalgamation of additive manufacturing with biomimicry shows great potential as a subject of future investigation for water collecting and may be potentially useful for relieving the water crisis in arid regions [55,56].

## Data Availability

Data will be made available upon request.

## Supplementary Materials

The following supporting information can be downloaded at: https://www.mdpi.com/article/10.3390/biomimetics9120734/s1, Table S1: Parameters of samples manufactured and tested. Figure S1: Experimental setup and Testing environment. Figure S2: Bionic simulation of 3D printed spine structures with effect temperature at different time intervals. Figure S3: Bionic simulation of 3D printed spine structures with effect Reynolds number at different time intervals. Figure S4: Bionic simulation of 3D printed spine structures with effect pressure at different time intervals. Figure S5: Bionic simulation of 3D printed spine structures with effect velocity at different time intervals.
