# Supplementary material for: Three-Dimensional Printing of Bioinspired Hierarchical Structures for Enhanced Fog Collection Efficiency in 3D Space via Vat Photopolymerization
Source: Biomimetics (Basel). 2024 Dec 3;9(12):734. doi: 10.3390/biomimetics9120734 (PMC11673100; doi:10.3390/biomimetics9120734)
Supplement: Supplementary file 1 [file biomimetics-09-00734-s001.zip › biomimetics-3281366-supplementary.pdf]

Supporting Information (Table and Figures)

Table S1. Parameters of samples manufactured and tested.

Sample #	Size, L_1 (µm)	Angle$_{xy}$, θ (°)	Angle$_{xz}$, ϕ (°)	Hierarchal Arrangement (AH / UH)	Number of Barbs, N_b	Number of Spines, N_s	Tested? (Y/N)
**1**	650	90	45	UH	1	1	**N**
**2**	760	90	45	UH	1	1	**N**
**3**	800	90	45	UH	1	1	**N**
**4**	900	90	45	UH	1	1	**Y**
**5**	900	120	45	UH	1	1	**Y**
**6**	900	45	45	UH	1	1	**Y**
**7**	900	45	45	AH	1	1	**Y**
**8**	900	45	45	AH	2	1	**Y**
**10**	900	45	45	AH	2	5	**Y**
**11**	900	45	45	AH	2	6	**Y**
**12**	900	45	45	AH	2	9	**Y**
**13**	900	45	75	AH	2	9	**Y**
**14**	900	45	90	AH	2	5	**Y**
**15**	1000	90	45	UH	1	1	**Y**
**16**	1150	90	45	UH	1	1	**Y**

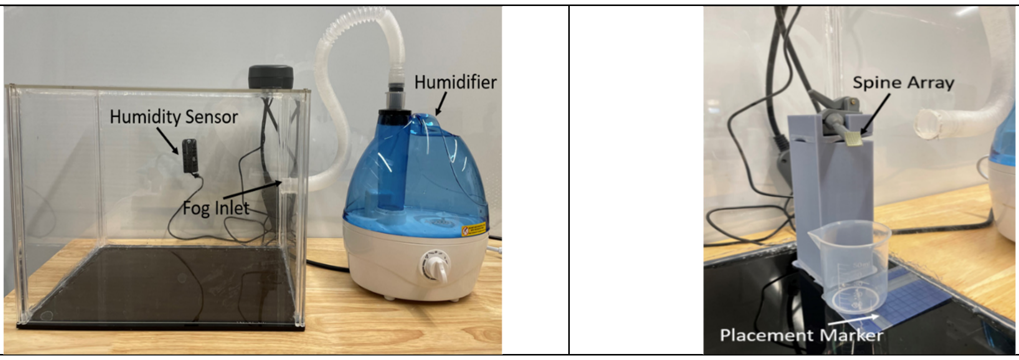

Figure S1. Experimental setup and Testing environment

Temperature\ Time	0 min	15 min	30 min	45 min	60 min
XZ angle 45	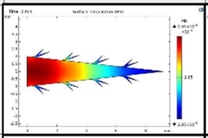	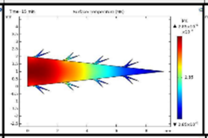	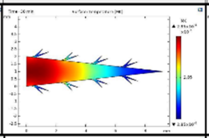	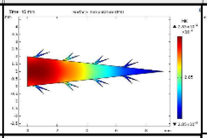	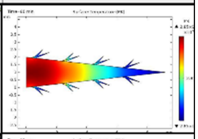
XZ angle 75	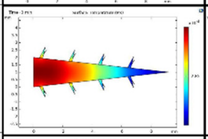	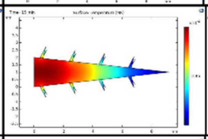	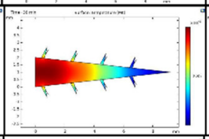	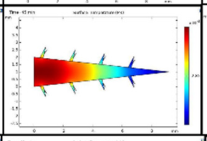	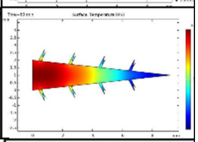
XZ angle 90	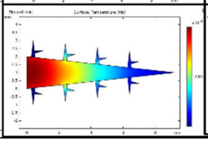	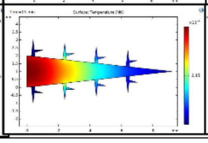	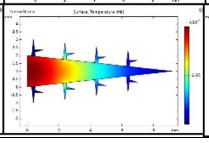	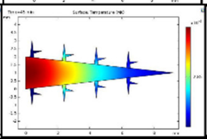	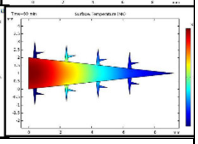

Figure S2. Bionic simulation of 3D printed spine structures with effect temperature at different time intervals.

Reynolds number\ Time	0 min	15 min	30 min	45 min	60 min
XZ angle 45	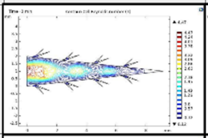	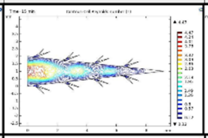	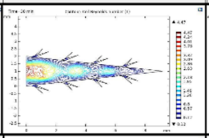	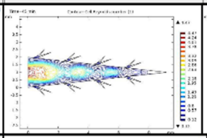	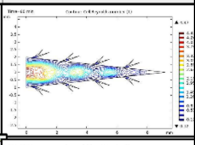
XZ angle 75	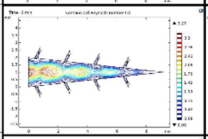	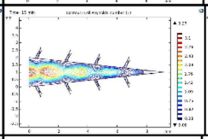	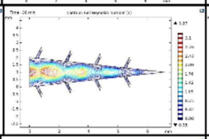	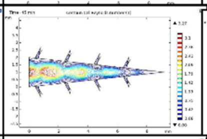	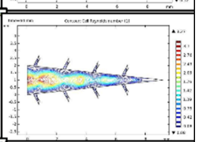
XZ angle 90	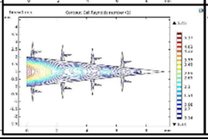	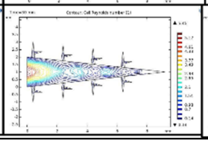	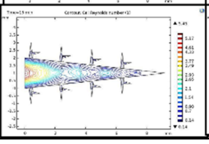	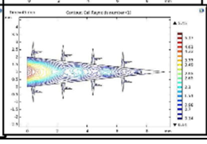	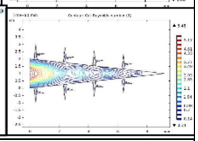

Figure S3. Bionic simulation of 3D printed spine structures with effect Reynolds number at different time intervals.

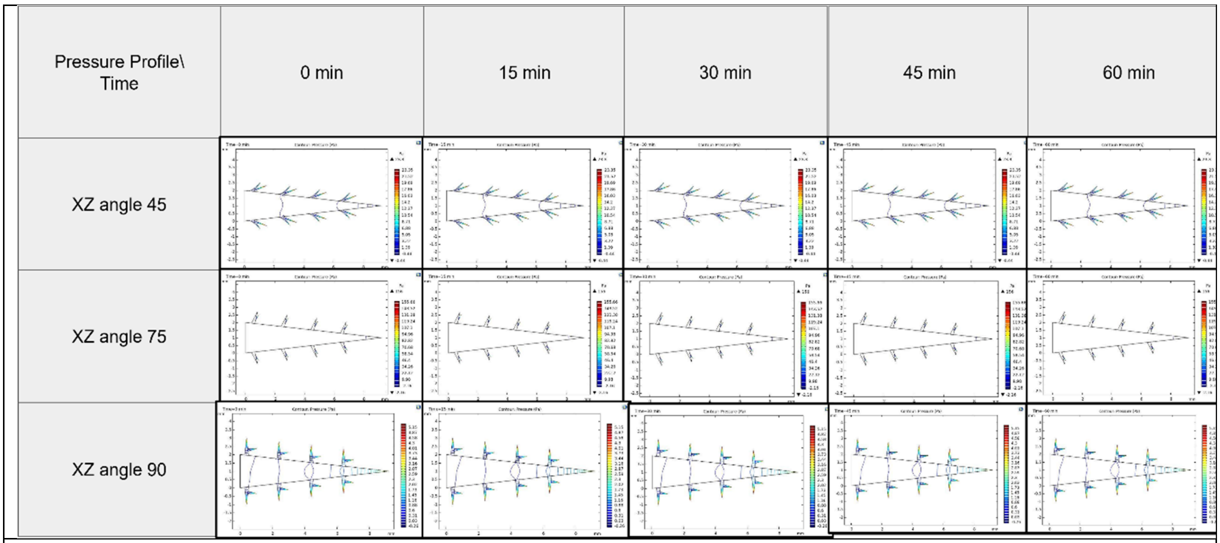

Figure S4. Bionic simulation of 3D printed spine structures with effect pressure at different time intervals.

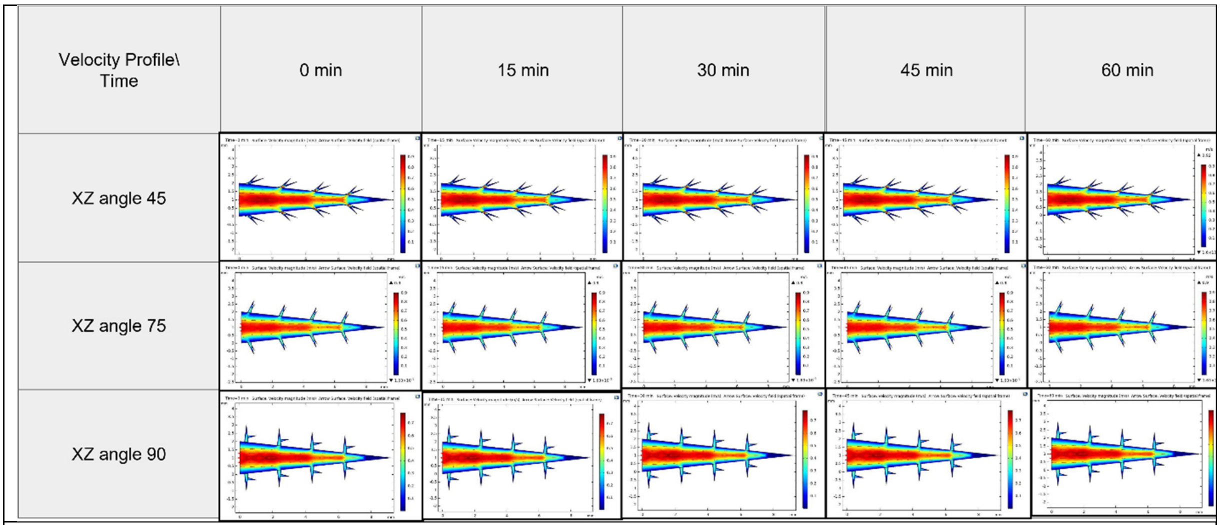

Figure S5. Bionic simulation of 3D printed spine structures with effect velocity at different time intervals.
